# A novel allele of the P-starvation tolerance gene *OsPSTOL1* from African rice (*Oryza glaberrima* Steud) and its distribution in the genus *Oryza*

**DOI:** 10.1007/s00122-014-2306-y

**Published:** 2014-04-13

**Authors:** Juan Pariasca-Tanaka, Joong Hyoun Chin, Khady Nani Dramé, Cheryl Dalid, Sigrid Heuer, Matthias Wissuwa

**Affiliations:** 1 Japan International Research Center for Agricultural Sciences (JIRCAS), 1-1 Ohwashi, Tsukuba, Ibaraki 305-8686 Japan; 2 International Rice Research Institute (IRRI), Los Banos, Laguna Philippines; 3 Africa Rice Center (Africa Rice), P.O. Box 33581, Dar es Salaam, Tanzania; 4 Australian Centre for Plant Functional Genomics (ACPFG), Hartley Grove, Urrbrae PMB 1, Glen Osmond, SA 5064 Australia

## Abstract

*****Key message***:**

**We have developed allele-specific markers for molecular breeding to transfer the** ***PSTOL1*** **gene from Kasalath to African mega-varieties, including NERICAs, to improve their tolerance to P-deficient soil.**

**Abstract:**

The deficiency of phosphorus (P) in soil is a major problem in Sub-Saharan Africa due to general nutrient depletion and the presence of P-fixing soils. Developing rice cultivars with enhanced P efficiency would, therefore, represent a sustainable strategy to improve the livelihood of resource-poor farmers. Recently the *Pup1* locus, a major QTL for tolerance to P deficiency in soil, was successfully narrowed-down to a major gene, the protein kinase *OsPSTOL1* (P-starvation tolerance), which was found to be generally absent from modern irrigated rice varieties. Our target is to improve the tolerance of African mega-varieties to P deficiency through marker-assisted introgression of *PSTOL1*. As a first step, we have determined the *Pup1* haplotype and surveyed the presence or absence of *PSTOL1* and other genes of the *Pup1* locus in African mega-varieties, NERICAs (New Rice for Africa) and their *Oryza glaberrima* parents. Here, we report the presence of a novel *PSTOL1* allele in upland NERICAs that was inherited from the *O. glaberrima* parent CG14. This allele showed a 35 base-pair substitution when aligned to the Kasalath allele, but maintained a fully conserved kinase domain, and is present in most *O. glaberrima* accessions evaluated. In-silico and marker analysis indicated that many other genes of the Kasalath *Pup1* locus were missing in the *O. glaberrima* genome, including the dirigent-like gene *OsPupK20*-*2*, which was shown to be downstream of *PSTOL1*. We have developed several allele-specific markers for the use for molecular breeding to transfer the *PSTOL1* gene from Kasalath to African mega-varieties, including NERICAs.

**Electronic supplementary material:**

The online version of this article (doi:10.1007/s00122-014-2306-y) contains supplementary material, which is available to authorized users.

## Introduction

The deficiency of phosphorus (P) in soil is a worldwide problem affecting about 50 % of the rice-cultivated area. Low P in soil may be due to the low P content of the parental material, low pH and/or soil with high P-fixing characteristics (Rose and Wissuwa 2012). In sub-Saharan Africa (SSA), many soils are characterized by deficient levels of plant-available P. Most of the soils in the semiarid zone were derived from acidic parent material that contained low levels of P (Buresh et al. 1997). For the once P fertile soils, soil P stocks have decreased as a constant population growth has led to continuous cropping on the same land without an adequate fertilization (Buresh et al. 1997; Sanchez 2002; Mokwunye and Bationo 2011). Annual average nutrient loss in SSA was estimated at 22 kg of nitrogen (N), 2.5 kg of phosphorus (P), and 15 kg of potassium (K) per hectare of cultivated land, which accounted for an annual loss equivalent to US $4 billion in fertilizer (Sanchez et al. 1997). These rates are several times higher than Africa’s annual fertilizer consumption, which accounts for only 0.8 % (1.29 Mt) of the global fertilizer consumption (International Fertilizer Development Center 2013). Thus, it would be very desirable, if not necessary, to enhance fertilizer use in much of Africa. The development of low-cost technologies for alleviating P deficiency, such as direct use of low-grade phosphate rock in conjunction with organic resources (Nakamura et al. 2013; Appleton 2002) could offer a sustainable solution.

Although P deficiency in soil could be alleviated through fertilizer application, the increasing price of fertilizer is becoming further prohibitive for resource-poor farmers in small scale farming systems (Rose et al. 2010). Approximately 10 % of the global population lives in Africa (International Fertilizer Development Center 2013), however, they use only 0.8 % (1.29 TM) of the total amount of applied fertilizer. Therefore, the development of cultivars with enhanced tolerance to P deficiency and other stress conditions would represent a parallel strategy to improve yield and enhance food security for rice.

Different genetic approaches have been taken to tackle this problem, and perhaps the most successful to date is the identification and characterization of the major quantitative trait locus (QTL) Phosphorus uptake 1 (*Pup1*). *Pup1* was identified in the rice variety Kasalath (Wissuwa et al. 2002) and near isogenic lines (NILs) carrying this QTL showed that *Pup1* conferred a significant yield advantage compared to the intolerant recurrent parent Nipponbare (Wissuwa 2005).

Sequencing the genomic region in Kasalath revealed a complex locus, including the presence of a ~90-kb transposon-rich insertion-deletion (INDEL) region that was absent from the genome of Nipponbare (Heuer et al. 2009). Recently, a large-effect gene was identified within *Pup1*, and the functional mechanism of this novel protein kinase-encoding gene (named P-STarvation TOLerance 1, *OsPSTOL1*) has been elucidated (Gamuyao et al. 2012). In addition, *Pup1* gene-specific molecular markers have been developed to assess the *Pup1* haplotype in diverse rice genotypes, and to improve the P-deficiency tolerance of Asian rice varieties (Chin et al. 2011). Using marker-assisted selection and backcrossing (MABC), the *Pup1* QTL region including the *OsPSTOL1* gene has been transferred into two Asian irrigated varieties (IR64-*Pup1* and IR74-*Pup1*), as well as into three Indonesian upland varieties (Chin et al. 2011). These breeding lines are now being evaluated in multiple locations across Asia (Chin et al. unpublished; Prasetiyono et al. unpublished).

Our ultimate goal was to introduce Os*PSTOL1* (hereafter referred to as *PSTOL1*) into African mega-varieties. Recent studies have shown that some popular upland NERICA (‘New Rice for Africa’) varieties with high yield potential (e.g., NERICA4) perform poorly under P-deficiency condition (Koide et al. 2013). Such varieties may thus benefit from the marker-assisted introgression of *PSTOL1*. Our preliminary work focused on the identification of suitable recipient varieties lacking the *PSTOL1* gene. Screening of several important African varieties using two *PSTOL1*-specific markers that were based on the Kasalath sequence (Chin et al. 2011) produced inconclusive results as weak, possibly unspecific, bands were amplified. We have sequenced those bands and report the presence of a novel *PSTOL1* allele in upland NERICAs that was inherited from the *O. glaberrima* parent, CG14. Based on this initial finding, *PSTOL1* allele-specific markers were developed and used for genotyping Asian and African rice cultivars and their ancestors. In addition, we have examined to what extent the entire *Pup1* locus is present in *O. glaberrima* and other *Oryza* species.

## Materials and methods

### Plant material

Seeds of rice varieties and wild *Oryza* species were obtained from IRRI, AfricaRice and JIRCAS germplasm bank. Seeds were surface sterilized with sodium hypochlorite, rinsed and incubated for 2–3 days at 30 °C. The germinated seeds were then transferred to a mesh floating on Yoshida nutrient solution [containing at full strength: N 2.86 mM (as NO_3_NH_4_), P 0.05 mM, K 1 mM, Ca 1 mM, Mg 1 mM, Mn 9 μM, Mo 0.5 μM, B 18.5 μM, Cu 0.16 μM, Fe 36 μM, Zn 0.15 μM]. The nutrient solution (half-strength) was replaced weekly, until leaf samples were taken at the 3rd week.

### DNA extraction

Small pieces of leaves tissue were flash-frozen in liquid nitrogen and kept at −80 °C until analysis. The frozen tissue was disrupted using a Qiagen mixer mill (Retsch MM 300, Germany), and tungsten carbide beads for 1 min at 25 pulses s^−1^. Afterwards, DNA was extracted using the DNeasy Plant Mini Kit (Qiagen), following the manufacturers protocol. In brief, the leaf tissue was homogenized in the presence of kit buffers, the homogenate was passed through spin columns and treated with RNase-A (Qiagen). DNA was eluted using TE buffer (10 mM Tris–HCl and 0.5 mM EDTA, pH 9.0), quantified by OD using a Nanodrop spectrophotometer (Thermo Scientific, USA), and DNA integrity was confirmed by electrophoresis in 2.0 % agarose gel.

### Genotyping

PCR reactions were performed using genomic DNA (25 ng), pairs of gene-specific primers, and Taq polymerase (Takara, Japan). PCR thermal conditions were as follows: first denaturing step at 94 °C for 2 min, followed by 30 cycles of 94 °C for 30 s, 55–60 °C for 30 s, and 72 °C for 90 s, and concluded by an extension step at 72 °C for 10 min. A part of the primer set used in this study was reported previously by Chin et al. (2010), including co-dominant markers K05, K20, K29-1, K29-3, and dominant markers K41, K42, K43, K45, K46-1, K48, K52 and K59 (located in the Kasalath-specific INDEL region). Polymorphism among the genotypes was detected by electrophoresis of the PCR products. Alleles were coded based on similarity to Kasalath (K), Nipponbare (N), CG-14 (CG), missing (M), or unknown genotype allele (U).

### Cloning, sequencing and alignments

The PCR products were gel purified using a spin-column (Promega, Madison), ligated into the pGEM-T Easy Vector (Promega, Madison), and the ligated product was used to transform Escherichia coli JM109 competent cells (Takara, Japan) following the manufacturer’s instructions. The plasmid DNA of positive clones was extracted using the PureYield™ Plasmid Miniprep System (Promega, Madison), and their sequence determined using the pUC/M13 forward or gene-specific primers. The amplicon identity was confirmed by nucleotide similarity using the Basic Local Alignment Search Tool (BLAST) software.

The *O. glaberrima* (IRGC accession # 96717, variety name CG14) genome sequence was obtained from the Arizona Genomics Institute of the University of Arizona (ftp://glabgenome@ftp.genome.arizona.edu/), and local BLAST was performed using the program BioEdit (http://www.mbio.ncsu.edu/bioedit/bioedit.html, version 7.1.11) and/or gramene (http://www.gramene.org/). Sequence alignment was performed using the MAFFT 7 software (http://mafft.cbrc.jp/alignment/server/index.html), and Jalview (http://www.jalview.org/).

### Transcript abundance analysis

In order to analyze the transcript abundance of the Kasalath and CG14 alleles, genotypes harboring each allele were selected and grown in soil or hydroponic culture.

Pots were filled with either P-deficient or P-fertilized soil as previously described by Pariasca-Tanaka et al. (2009). Seeds were sown directly in the soil to facilitate normal root development, and watering was carried out regularly to simulate upland condition. In hydroponic experiments, pre-germinated seeds were placed on a floating mesh for 1 week, and afterwards seedlings were transferred to 12-L containers with Yoshida nutrient solution (as described above) at two P-treatments: 2 µM P (low P) or 50 µM P (control, sufficient P). Tissue samples were taken 40 days after sowing (DAS); shoots (leaf blades) and roots were rapidly frozen in liquid nitrogen and stored at −70 °C until analyzed.

### RNA extraction and RT-PCR

Total RNA was extracted using the RNeasy Mini Kit (Qiagen, USA) according to the manufacturer’s instruction as previously described by Pariasca-Tanaka et al. (2009). Total RNA (around 400 ng) was reverse transcribed (RT) using OligodT and Random 6-monomer primers, and a PrimeScript RT Enzyme Mix I (Takara, Japan) at 37 °C for 15 min, followed by a inactivation of the enzyme at 85 °C for 5 s, and storage at 4 °C.

Subsequently, allele-specific primers, Taq polymerase (Takara, Japan), and the first-strand cDNA were used for PCR. PCR thermal condition was as follows: first denaturing step at 94 °C for 2 min, followed by 30 cycles of 94 °C for 30 s, 55–60 °C for 30 s, and 72 °C for 90 s, and concluded by an extension step at 72 °C for 10 min.

## Results

**Fig. 1:**
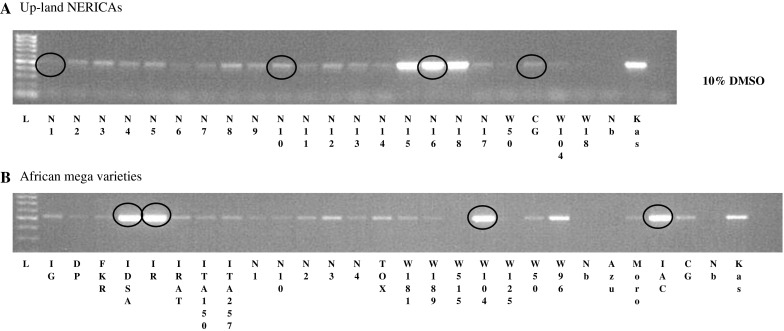
Amplification of unspecific bands when *PSTOL1*-specific marker (K46-1, based on Kasalath sequence) was used for PCR genotyping of upland NERICA varieties and their parents (**a**); and upland African mega-varieties (**b**). DMSO was added to improve PCR amplification. *Red circles* indicate the amplicon of genotypes selected for sequencing (color figure online)

Gamuyao et al. (2012) showed that the protein kinase *PSTOL1* is the major gene responsible for enhanced P uptake conferred by the *Pup1* locus. To survey the presence of *PSTOL1* in NERICAs and other important African varieties, we have initially used *PSTOL1*-specific PCR-based molecular markers that were designed based on the sequence of the *Pup1* donor variety Kasalath (Chin et al. 2011). Although the amplified DNA fragments had the expected size (523 bp) in all samples analyzed, strong bands were obtained in only a few samples, the majority showing weak bands (Fig. 1). This experiment was repeated several times with inconsistent results (data not shown). Therefore, several of these weak bands were sequenced and the sequences aligned with the Kasalath *PSTOL1* (*OsPupK46*-*2*). The Kasalath amplicon, which was included as a positive control, showed 100 % sequence identity with the Kasalath sequence deposited in the gene bank with Acc. number AB458444 (Online Resource 1—Fig.S1). Of the 11 additional genotypes sequenced, four aligned perfectly to the Kasalath sequence. This included WAB56-104 (short: W104), the parent of NERICA1 to NERICA11. In contrast, the sequence of CG14, the *O. glaberrima* parent of NERICAs, had 14 nucleotide substitutions relative to the Kasalath sequence. Several of the tested NERICAs (N1, N2, N4, N6, N10) shared most substitutions with CG14, suggesting the presence of an *O. glaberrima*-specific *PSTOL1* allele that NERICA varieties had inherited from CG14. However, a few nucleotide substitutions were only detected in CG14 (confirmed against the CG14 DNA database, see below) and in one or two NERICAs (Online Resource 1—Fig.S1). These may represent recent mutations in the second *PSTOL1* allele or slight differences between parental accessions used in this study and in making the crosses resulting in NERICA varieties.

### Comparison between *PSTOL1* and the *O. glaberrima* gene model

The presence of an *O. glaberrima*-specific *PSTOL1* allele was verified in the *O. glaberrima* (CG14) genome sequence obtained from the Arizona Genomics Institute (AGI) website (*Oryza glaberrima* genome assembly version 1). The CG14 sequence had been assembled against the Nipponbare reference genome, which lacks *PSTOL1* and the adjacent sequences of a large (~90 kb) *Pup1*-specific insertion–deletion. It therefore was not surprising that *PSTOL1* was detected in an unanchored scaffold (Oglab12_unplaced142) derived from the chromosome 12 pool 6 (96 % sequence identity), rather than in assembled CG14 genome sequence.

**Fig. 2:**
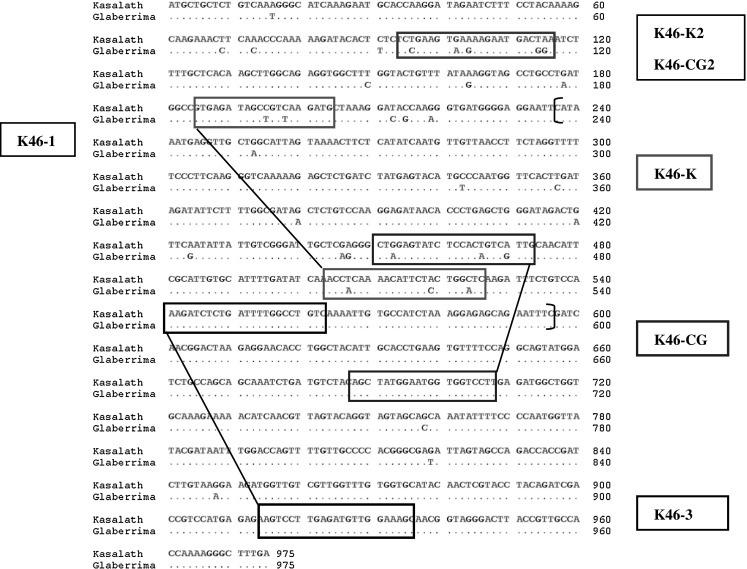
Nucleotide sequence alignment of the *PSTOL1* allele from Kasalath (*O. sativa*, ssp. indica) and CG14 (*O. glaberrima*). *Brackets* indicate the region sequenced from the amplicons. Polymorphic SNPs are indicated. *Squares* indicate the location of allele-specific markers for *PSTOL1* alleles of Kasalath and CG14. Location of *PSTOL1* allele in *O. glaberrima*: 116753–115779, Oglab12_unplaced142#*O. glaberrima* unanchored scaffold derived from chr12 pool6 (represented by Oglab12_0135 thru Oglab12_0185). Precise location and orientation is unknown (Arizona Genome Institute)

A sequence comparison between *PSTOL1* with the corresponding CG14 gene (position: 116753–115779 bp on ‘Oglab12_unplaced142’) revealed 35 nucleotide substitutions within the 975-bp sequence (Fig. 2, Online Resource 2—Fig.S2), confirming the presence of an *O. glaberrima PSTOL1* allele.

### Development of duplex PCR

**Fig. 3:**
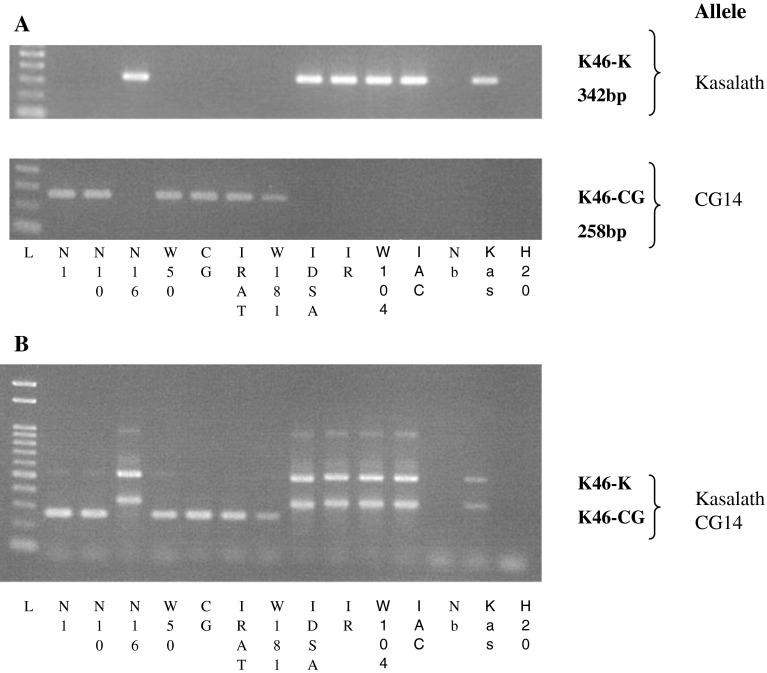
PCR amplification for *PSTOL1* using Kasalath (*O. sativa*, ssp. indica) and CG14 (*O. glaberrima*) allele-specific markers (**a**). The mixture of these two pairs of allele-specific markers resulted in a duplex-PCR genotyping method (**b**). (*N1* NERICA1, *N10* NERICA10, *N16* NERICA16, *W50* WAB56-50, *CG* CG14, *IRAT* IRAT 216 (IDSA6), *W181* WAB181-18, *IDSA* IDSA 85, *IR* 12979, *W104* WAB56-104, *IAC* IAC165, *Nb* Nipponbare, *Kas* Kasalath)

As outlined above, our initial screening of potential recipient cultivars was hampered by the erratic presence of weak bands during PCR amplification with *PSTOL1* primers. This can now be explained by the presence of two nucleotide substitutions in the CG14 allele located within the binding site for the forward primer of the marker ‘K46-1’ (Fig. 2). New primers were, therefore, designed specifically targeting nucleotide polymorphisms between the two *PSTOL1* alleles (Fig. 2; Table 1). Best results were obtained with primer pairs K46-K, which specifically amplifies the Kasalath *PSTOL1* allele, and with primer pair K46-CG, specific for the CG14 allele (Fig. 3a). Additional allele-specific primers were developed (Table 1), which may be useful for the genotyping of specific genetic backgrounds, or if amplicons of different sizes are required.

**Table 1:** Design of Kasalath and CG14 allele-specific markers for genotyping diverse genotypes

Marker name	Allele	Sequence (5′–3′)		Size (bp)
K46-1^a^	Kas	K46-1	K46-1	523
		Fw: TGAGATAGCCGTCAAGATGCT	Rv: AAGGACCACCATTCCATAGC	
K46-K1	Kas	K46-1	K46-Ksp3rv	342
		Fw: TGAGATAGCCGTCAAGATGCT	Rv: TGAGCCAGTAGAATGTTTTGAGG	
K46-CG1	CG	K46-CGsp2fw	K46-1	258
		Fw: CTAGAGTATCTCCACAGTCGTT	Rv: AAGGACCACCATTCCATAGC	
K46-K2	Kas	K46-Ksp4fw	K46-Ksp3rv	433
		Fw: CTGAAGTGAAAAGAATGACTAA	Rv: TGAGCCAGTAGAATGTTTTGAGG	
K46-CG2	CG	K46-CGsp4fw	K46-CGsp3rv	433
		Fw: CCGAAGTAAGAAGAATGACGGA	Rv: TGATCCAGGAGAATGTTTTGTGG	
K46-3	Kas/CG	K46-3	K46-3	400
		Fw: TCCAAAGATCTCTGATTTTGGC	Rv: GCTTTCCAACATCTCAAGGACT	

^a^Chin et al. 2011

One additional feature of the developed allele-specific markers is that they can be combined in a duplex PCR. The size difference of the amplicons is sufficiently large (342 vs. 258 bp) to differentiate the distinct *PSTOL1* alleles in a single PCR reaction and subsequent gel electrophoresis (Fig. 3b). A single band (258 bp) is indicative of the CG14 allele, whereas the Kasalath *PSTOL1* allele is indicated by the expected 342-bp amplicon and a second amplicon (~500 bp) derived from the K46-1 primer pair that is reconstituted in the duplex assay. Other primer combinations did not produce clear diagnostic band pattern (data not shown).

In addition to the allele-specific markers, we have designed marker K46-3, which targets a conserved region (Fig. 2; Table 1) and amplifies both *PSTOL1* alleles equally (Online Resource 3—Fig.S3).

### Assessing the presence of *PSTOL1* alleles across cultivated and wild rice

The duplex marker system was further employed to determine the *PSTOL1* allele across a wide selection of cultivated and wild *Oryza* accessions. A complete list of the accessions tested and the genotyping results are given in Online Resource 4 (Table S1). Among the 76 *O. sativa* accessions tested, *PSTOL1* was absent from 26 accessions, whereas 23 accessions possessed the Kasalath and 27 possessed the CG14 allele (Table 2, Online Resource 4—Table S1-*O. sativa*). Within the *O. sativa* sub-groups, *indica* type most commonly lacked the *PSTOL1* gene, whereas *aus*-type accessions mainly had the Kasalath allele. Tropical *japonica* accessions were common in all three groups (Online Resource 4—Table S1-*O. sativa*).

**Table 2:** Distribution of *OsPSTOL* allele in the genus *Oryza*

Species	Genome	*n*	Allele			
			K	CG	Novel	Absent
*O. sativa*	AA	76	23	27	–	26
*O. glaberrima*	AA	44	2	39	3	–
*O. nivara*	AA	6	2	1	2	1
*O. rufipogon*	AA	10	3	1	3	3
*O. longistaminata*	AA	6	5	–	1	–
*O. barthii*	AA	10	1	9	–	–
*O. glumaepatula*	AA	3	1	–	–	2
*O. meridionalis*	AA	3	–	2	–	1
*O. punctata*	BB	5	–	–	–	5
*O. officinalis*	CC	11	–	–	–	11
*O. australiensis*	EE	4	–	–	–	4
*O. brachyantha*	FF	1	–	–	–	1
*O. granulata*	GG	4	–	–	–	4
*O. minuta*	BBCC	5	–	–	–	5
*O. alta*	CCDD	4	–	–	–	4
*O. grandiglumis*	CCDD	2	–	–	–	2
*O. ridleyi*	HHJJ	1	–	–	–	1

PCR was performed using allele-specific markers for genotyping

In contrast, *O. glaberrima* accessions (44) predominantly had the CG14 allele, with only two accessions showing the Kasalath allele and three having no amplification (Table 2, Online Resource 4—Table S1-*O. glaberrima*). The *O. glaberrima* CG14 allele was also predominantly present in interspecific NERICA upland accessions, but mostly absent in lowland NERICA (NERICA L) accessions with the exception of the group NERICA L23, L24, and L25, which all contained the CG14 allele (Online Resource 4—Table S1-NERICA).

Surprisingly, NERICA15, 16 and 18 varieties showed the Kasalath allele despite both parents (CG14 and WAB181-18) lacking the CG14 allele. Similarly, NERICA L15, L28, L29 and others showed the Kasalath allele despite this allele being absent in both parents (TOG5681 has the CG14 allele while IR64 completely lacks the gene). These unexpected genotypes may be explained by the presence of non-parental introgressions, which have been reported in NERICAs (Semagn et al. 2007; Koide et al. 2013). We also detected inconsistencies in WAB56-104, the *sativa* parent of NERICA1–11: the accession from AfricaRice showed the Kasalath allele, but a different accession in use at JIRCAS was genotyped as having the CG14 allele (Online Resource 4—Table S1-*O. glaberrima*). Among the important African rice varieties, the majority had the CG14 allele and only one cultivar (WAB515-B-16-A2-2) lacked *PSTOL1* (Online Resource 4—Table S1-*O. sativa*).

Additionally, a set of 76 wild rice accessions was genotyped using the *PSTOL1* allele-specific markers. Within *O. nivara* and *O. rufipogon*, the ancestors of *O. sativa*, no consistent genotype was detected since the *PSTOL1* gene was either absent or present as Kasalath or CG14 allele (Table 2, Online Resource 4—Table S1-wild rice). In addition, a putative novel allele was detected which was amplified with both, the Kasalath and CG14-specific markers (Online Resource 4—Table S1-wild rice). This allele was also detected in *O. longistaminata* (Table 2), but not in *O.* *barthii*, the *O. glaberrima* ancestor. Interestingly, nine out of ten *O. barthii* accessions showed amplification with the *O. glaberrima* CG14 allele-specific marker. The presence of *PSTOL1* was restricted to *Oryza* species belonging to the AA genome, since no allele could be detected in accessions with BB ~ HHJJ genomes (Online Resource 4—Table S1-wild rice).

### Transcript abundance

**Fig. 4:**
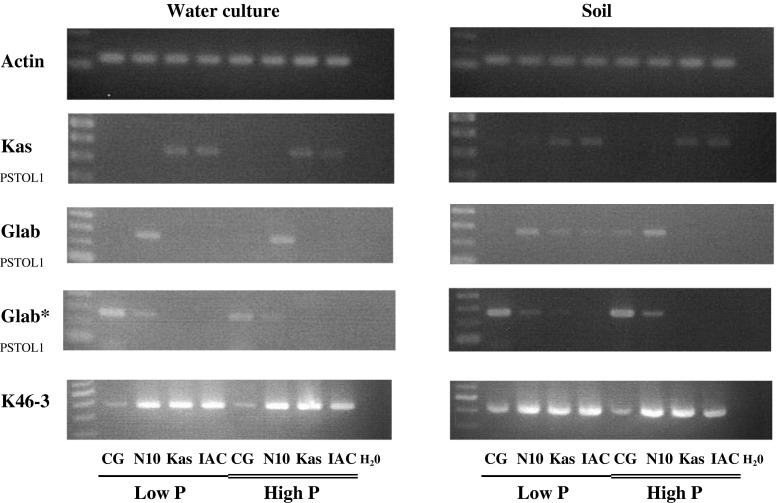
Abundance pattern of *PSTOL1* transcript in four rice genotypes grown in water culture or soil under low or high P condition. Specific-allele markers for *PSTOL1*-Kasalath (*O. sativa*) or *O. glaberrima*, and K46-3 (which amplifies a common region in both alleles) were used for RT-PCR. *CG* CG14, *N10* NERICA10, *IAC* IAC165, *Kas* Kasalath. Glab* cycles were increased up to 40, and template doubled, only for CG14

Genotypes harboring the Kasalath allele (Kasalath, and IAC165) or the CG14 allele (CG14 and NERICA10) were grown in P-deficient and P-replete conditions to determine their *PSTOL1* transcript abundance. An RT-PCR analysis using total RNA extracted from roots and allele-specific and unspecific primers confirmed the existence of the CG14 allele at the transcript level (Fig. 4). Expression of both alleles appeared to be constitutive and not noticeably enhanced by P deficiency. The abundance of the CG14 transcript in NERICA10 was comparable to that of the Kasalath allele (Kasalath and IAC165) in both water culture and soil. Interestingly, *PSTOL1* transcript abundance was very low in CG14, irrespective of the growth conditions (Fig. 4). The results obtained with the allele-specific markers were also confirmed with the marker K46-3, which is located in the conserved region (Fig. 4). Furthermore, in-silico analysis of the protein sequence indicated that the kinase catalytic domain (RIVHFDIKPQNILL) was conserved in the CG14 allele (Online Resource 5—Fig. S4). Overall amino acid sequence similarity between both alleles was 94.1 %.

### Comparison of the entire *Pup1* locus

**Fig. 5:**
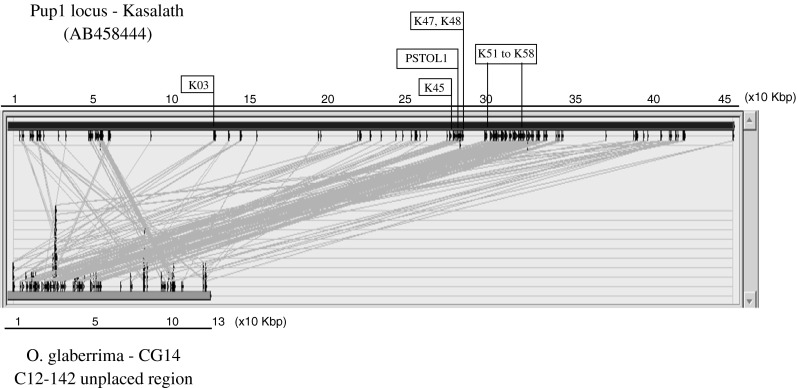
Sequence alignment between the Kasalath *Pup1* region (AB458444) and a region containing the *PSTOL1* allele in the Chromosome 12-unplaced 142, of *O. glaberrima* (Arizona Genome Institute)

The presence of a novel *PSTOL1* allele in *O. glaberrima* raised the question to what extent the entire Kasalath *Pup1* locus (AB458444) is conserved in *O. glaberrima* (CG14). A sequence alignment of the *Pup1* locus against the CG14 scaffold-containing *PSTOL1* (Glab 12_unplaced 142#; AGI) showed high sequence similarity for about 30 % of the 130-Kbp length. Kasalath gene models *OsPupK45* to *OsPupK48* and *OsPupK51* to *OsPupK58* were detected in the *O. glaberrima* sequence in addition to *PSTOL1* (Fig. 5). These genes are located in the Kasalath *Pup1*-specific INDEL region which is absent from the Nipponbare reference genome. No other large scaffold was found to align with *Pup1*.

Subsequently, a BLAST search for each of the putative 68 *Pup1* gene models indicated that only the genes *OsPupK01*-*1* and *OsPupK67*-*1* (located at the very 3′- and 5′-end of the *Pup1* region) are present in the assembled *O. glaberrima* sequence. They were located on Chr12 at 11.683 and 13.578 Mbp, respectively (Gramene). In contrast, *PSTOL1* and the other genes located in the INDEL (see above), as well as other relevant *Pup1* genes are not present in the assembled *O. glaberrima* genome sequence.

**Fig. 6:**
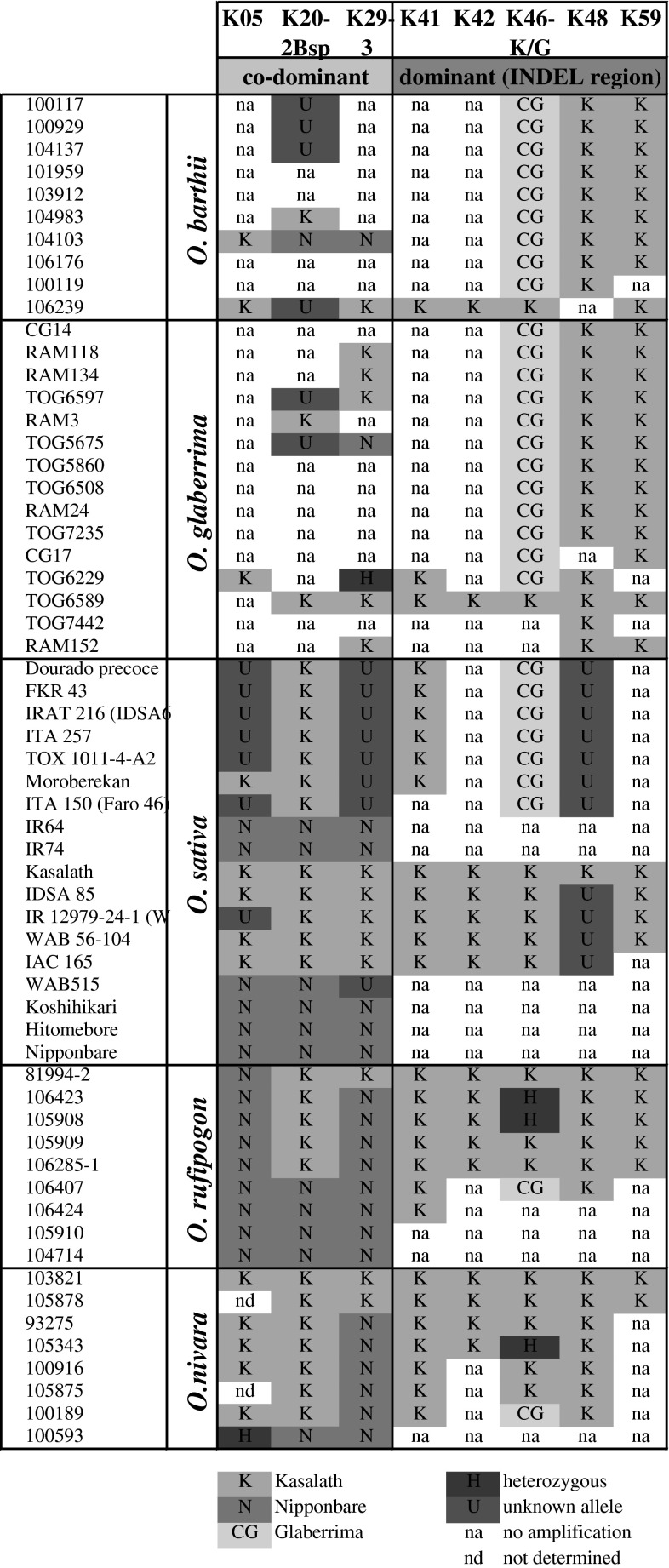
Presence of several co-dominant (K20-2 and K29-3) and dominant (K41, K42, *PSTOL1*, K48, K29) *Pup1* markers in wild genotypes of rice

Whereas the former is likely due to difficulties with the anchoring and assembly of the genome, absence of relevant genes located in partially conserved *Pup1* regions (e.g., *OsPupK05*, *OsPupK20*-*2* and *OsPupK29*-*1*; Gamuyao et al. 2012) might indicate real and significant structural differences between the *Pup1* region in *O. glaberrima* and *O. sativa.* Indeed, absence of *OsPupK05*, *OsPupK20*-*2* and *OsPupK29*-*1* from the genome of CG14 and the majority of *O. glaberrima* accessions were subsequently confirmed by PCR analyses using gene-specific markers (Fig. 6). The fact that *O. barthii* is also lacking these genes, whereas some *O. nivara* and *O. rufipogon* accessions have them is suggesting that the presence/absence of part of *Pup1* can be traced to wild ancestors.

## Discussion

Demand for rice by consumers in SSA is rapidly increasing and continues to outpace local rice production. Annual per capita consumption of milled rice in SSA increased from 14 kg in 1970 to more than 39 kg in 2009 resulting in importation of nearly 40 % of the rice consumed in the region (Seck et al. 2012). Enhancing rice production on the African continent, therefore, is a goal of both national governments and international organizations. Different partnership programs such as the Coalition for African Rice Development (CARD) established in 2008 (TICAD IV) and the Global Rice Science Partnership (GRiSP) of CGIAR in 2010 are working towards this end. As rice cultivation expands in area, adaptations to low soil fertility will become increasingly important, particularly since the combination of widespread occurrence of poor soils and low fertilizer application rates remains a challenge throughout much of Africa (Sanchez 2002). In response to this challenge, the objective of JIRCAS and AfricaRice (both involved in GRiSP) is to adapt popular African varieties (mega-varieties) to low soil fertility using modern breeding techniques such as marker-assisted selection (MAS).

NERICA varieties are popular in the uplands of West and East Africa (Jones et al. 1997), and a new set of interspecific hybrid lowland NERICAs has great potential in lowland ecologies (Sié et al. 2005). Some of the most successful upland NERICAs (e.g., NERICA4) have high yield potential when soil fertility is high, but they lack adaptation to P deficiency (Koide et al. 2013). One may therefore expect that they would benefit from the marker-assisted introgression of QTLs such as *Pup1* that enhances P-deficiency tolerance. A similar strategy to enhance P-deficiency tolerance in Asian rice cultivars is being followed and the first results confirm that the introgression of *PSTOL1* (*Pup1*) through marker-assisted backcrossing (MABC) can enhance root growth and subsequent P uptake of the Asian lowland rice variety IR74 (Gamuyao et al. 2012). While the marker diagnostic of *PSTOL1* (K46-1, Chin et al. 2011) is mainly used in such MABC schemes, it should be noted that chromosomal segment introgressed typically encompasses the entire *Pup1* locus of Kasalath, or even a larger chromosomal region, depending on the design and placement of flanking markers.

Marker K46-1 is a dominant marker and highly diagnostic for crosses of the *Pup1* donor variety Kasalath with most Asian lowland rice varieties (e.g., Nipponbare, IR64, IR74, etc.,) because the *PSTOL1* gene is completely absent from these varieties (Chin et al. 2010, 2011). The situation is different for many African mega-varieties and NERICAs in which the presence of a novel *PSTOL1* allele caused unreliable genotypic data using this marker.

### Distribution, occurrence and possible origin of the novel PSTOL1 allele

Initial evidence for the existence of a novel *PSTOL1* allele derived from the K46-1 marker data in several NERICA accessions and the *O. glaberrima* parent CG14, as well as from in-silico comparison with the CG14 genomic sequence. Since the *O. sativa* parent (WAB56-104) of the analyzed NERICAs had the Kasalath allele, we concluded that NERICAs inherited the novel *PSTOL1* allele from CG14, which therefore likely originated from *O. glaberrima*. That 90 % of all *O.* *glaberrima* accessions tested also amplified the novel allele seemed to confirm this assumption, as did the fact that the same was true for 90 % of *O. barthii* accessions (wild ancestor of *O. glaberrima*). However, subsequent marker analyses led to the detection of the CG14 allele in 35 % of *O. sativa* accessions as well, and even few wild ancestors of *O. sativa* (*O. nivara* and *O. rufipogon*) amplified the CG14 allele. This allele therefore is the common allele of *O. glaberrima*, but it cannot be considered *O.* *glaberrima*-specific.

To further elucidate the origin of *PSTOL1* and the entire *Pup1* locus, we examined polymorphic patterns for several markers designed based on gene models present within the *Pup1* locus. These either originated from the INDEL region not present in Nipponbare (markers K41, K42, K46-K/G, K48, K59), or from a region common for both Kasalath and Nipponbare (markers K05, K20-2, K29-1). Generally, *O. glaberrima* and wild ancestor *O. barthii* had almost identical patterns with a partial presence of the Kasalath INDEL region. Ancestors of *O. sativa*, on the other hand, mirrored the contrast between Nipponbare and Kasalath: the most common pattern was the complete presence or absence of the INDEL region (Fig. 6).

These patterns suggest the divergence at the *Pup1* locus to be ancient, possibly pre-dating the domestication of *O. sativa* and *O. glaberrima*. The hypothesis that *PSTOL1* and surrounding INDEL was lost from *O. sativa* during the recent development of high-yielding modern semi-dwarf varieties, and that this loss may have been facilitated by the absence of nutrient deficiencies in well-fertilized modern breeding nurseries, is not supported by the present data. Unfortunately, the wild ancestor accessions studied lack the eco-physiological data to determine whether growth conditions at their origin would associate with the polymorphic patterns observed for the *Pup1* locus. It is therefore not possible to further investigate possible reasons for the diversions observed at *Pup1*.

### Implications for MAS

The presence of the novel CG14 allele in most African rice varieties raises the question as to whether it confers the same phenotypic advantage as the Kasalath allele. NERICA varieties differ in their tolerance to P deficiency, and the main QTL associated with tolerance has recently been mapped to chromosomes 4, 6 and 11 (Koide et al. 2013). The *Pup1* region on chromosome 12 had no effect in the QTL mapping, nor did it associate with differences between NERICA varieties (Wissuwa, unpublished). The data presented here provide evidence for why this may be, as there simply is no variation within NERICAs of same parental origin: NERICA1-11 (derived from WAB56-104) and NERICA12-14 (derived from WAB56-50) all carry the CG14 allele. Only NERICA15-18 shows variation, with NERICA17 carrying the CG14 allele and others the Kasalath allele, which indicates the presence of non-parental introgressions, as their *O. sativa* parent (WAB18-181) also carries the CG14 allele. The presence of either allele was not linked to their performance under P deficiency: NERICA17 and 18 were tolerant and NERICA15 sensitive (Koide et al. 2013). However, this does not allow for clear conclusions regarding the phenotypic effects of *PSTOL1* alleles, especially as NERICA17 and 18 differ for the presence of additional QTL on chromosomes 6 and 11.

The fact that the CG14 allele is expressed and that the functional domain is conserved may indicate the allele retained its function. However, several NERICA varieties (e.g., NERICA 4) perform poorly under P deficiency, while CG14 is very tolerant (Koide et al. 2013). One potential explanation is that other genes of the *Pup1* locus may also have a positive effect or interact with *PSTOL1* to produce the tolerant phenotype. The dirigent gene *OsPupK20* is one possible candidate because (i) it is missing in most *O. glaberrima*, and (ii) expression profiling in transgenic lines overexpressing *PSTOL1* indicated *OsPupK20* expression was upregulated in the transgenics (Gamuyao et al. 2012). *OsPupK20* may thus act downstream of PSTOL1, and could contribute to the final positive phenotype. Work has started to introgress the entire *Pup1* locus into NERICA4, and once advanced backcross generations become available, the question of allelic effects versus effects of other genes at the locus can be studied in more detail.

In the absence of clear evidence regarding functionality of different *PSTOL1* alleles and with a highly variable background locus, two questions arise with regard to the preferred MAS strategy: (1) can varieties other than Kasalath serve as suitable donors, and (2) will it be enough to only select for *PSTOL1* as the sole foreground marker?

We believe the preferred MAS breeding strategy should be to introgress the entire *Pup1* locus from Kasalath or a breeding line produced elsewhere from a Kasalath-based *Pup1* introgression scheme. Several such lines in IR64 or IR74 backgrounds have been developed at IRRI and are publicly available through the IRRI Genetic Resources Center (GRC). Using such lines as donors ensures that the entire *Pup1* locus is introgressed. Furthermore, a single diagnostic marker (e.g., K46-K) would suffice as a foreground marker.

However, compelling reasons for the use of *Pup1* donors other than Kasalath may exist. In that case, an initial genotyping step would have to establish to what extent the *Pup1* locus is present in the donor and recipient parents. The highly variable nature of *Pup1* likely requires at least four additional markers to obtain a reasonably good estimate of *Pup1* locus divergence in parents. If unspecific marker K46-3 had been used as the only marker, most *O.* *glaberrima* and several *O.* *sativa* would have appeared as suitable *Pup1* donors even though they possess a different *PSTOL1* allele and in many cases an entirely different *Pup1* locus.

The presence of a novel allele of unknown effect has furthermore highlighted a conceptual issue in MAS. It is frequently assumed that identifying the gene underlying a QTL is an unnecessary ‘luxury’, since markers diagnostic of the donor regions should suffice to introgress the donor segment at a QTL. While this is technically correct, the present example shows that a more detailed knowledge of the locus structure and candidate gene(s) is crucial to avoid two potential blunders in MAS: (i) the introgression of a locus/gene into a recipient that already possesses the same allele, and (ii) the choice of a novel donor based on phenotype without assuring it has the desired allele. Early examples of successful application of MAS in rice focused on very strong QTLs, such as the *sub1* locus for submergence tolerance, that in fact resembles a single gene or qualitative trait. In that case, the presence or absence of the QTL in recipient parents can be inferred directly from phenotyping. This would not be as straightforward for multigenic, truly quantitative traits, for which a single QTL may only explain 20–25 % of the variation. In such a situation, all but the most extreme phenotypes could be caused by the QTL in question or by background variation. Only markers that query the underlying gene or allele will diagnose the presence of the desired genotype reliably and more effort may need to be directed to identifying those.

## Electronic supplementary material

Below is the link to the electronic supplementary material.

Supplementary material 1 contains Online Resource 1, 2, 3 and 5 (DOC 395 kb)

Online Resource 4 - Table S1. Genotyping of *PSTOL1* alleles in *O. sativa* and *O. glaberima* accessions, NERICAs, several African mega-varieties, and wild relatives (XLS 64 kb)
