# Supplementary material for: A novel allele of the P-starvation tolerance gene OsPSTOL1 from African rice (Oryza glaberrima Steud) and its distribution in the genus Oryza
Source: Theor Appl Genet. 2014 Apr 13;127(6):1387–98. doi: 10.1007/s00122-014-2306-y (PMC4035548; doi:10.1007/s00122-014-2306-y)
Supplement: Supplementary file 1 — Supplementary material 1 contains Online Resource 1, 2, 3 and 5 (DOC 395 kb) [file 122_2014_2306_MOESM1_ESM.doc]

Online Resource 1 – Fig.S1. Alignment of the amplicon sequences from Kasalath (*O. sativa*, var indica), *O. glaberrima*, and several other genotypes. Amplicons were sequenced and aligned using ClustalW.

(Kasalath, IDSA, IAC65, IR12979, W104: WAB56-104, N1: NERICA1, N2: NERICA2, N4: NERICA4, N6: NERICA6, N10: NERICA10, N16: NERICA16, CG14)

Online Resource 2 – Fig.S2. Nucleotide sequence alignment of the *PSTOL1* allele from Kasalath (*O. sativa,* ssp indica) and *O. glaberrima*. Dots refer to similar nucleotide, while different nucleotide refers to polymorphic SNPs. Brackets indicate the region sequenced (Figure 2).

Location of *PSTOL1* allele in *O. glaberrima*: 116753 – 115779, Oglab12_unplaced142#O. glaberrima unanchored scaffold derived from chr12 pool6 (represented by Oglab12_0135 thru Oglab12_0185). Precise location and orientation is unknown (Arizona Genome Institute).

Online Resource 3 – Fig.S3. PCR amplification of the marker K46-3 in genomic DNA of Kasalath and CG14 (*O. glaberrima*). This marker is located in a common region for both alleles (400 bp).

K: Kasalath, CG: CG14, Nb: Nipponbare, W18: WAB181-18, W50: WAB56-50, IR64.

Online Resource 5 – Fig S4. Alignment of the deduced amino acid sequence for Kasalath and CG14 alleles. Red rectangular region refers to the kinase active site. Dots indicated similar base.
